# Technology Access and Preferences for Smartphone App Interventions to Optimize Iron Chelation Therapy Adherence Among Adolescents, Young Adults, and Parents of Children Receiving Chronic Transfusions: Cross-Sectional Survey Study

**DOI:** 10.2196/69584

**Published:** 2026-03-24

**Authors:** Paavani Reddy, Margaret Locke, Sherif Badawy

**Affiliations:** 1 Department of Psychiatry, Massachusetts General Hospital, Boston, MA, United States; 2 Department of Psychiatry, McLean Hospital, Belmont, MA, United States; 3 Department of Hematology and Oncology, Donald & Barbara Zucker School of Medicine at Hofstra/Northwell, Hempstead, NY, United States; 4 Division of Hematology, Oncology, and Stem Cell Transplant, Lurie Children's Hospital, 225 E Chicago Ave Box #30, Chicago, IL, United States, 1 312-227-4836, 1 312-227-9376; 5 Department of Pediatrics, Northwestern University Feinberg School of Medicine, Chicago, IL, United States

**Keywords:** thalassemia, sickle cell disease, medication adherence, iron chelation therapy, behavior change, technology access, mobile health, mHealth

## Abstract

**Background:**

Iron chelation therapy (ICT) is essential for people with hematological disorders requiring chronic transfusions to minimize the risk of iron overload, yet suboptimal adherence is prevalent. Widespread use of personal technology makes mobile health (mHealth) an attractive platform to promote adherence.

**Objective:**

This study aimed to examine access to mobile technology and preferences for an mHealth intervention to improve adherence to ICT.

**Methods:**

A cross-sectional survey that included 63 items assessing technology access, mHealth preferences, and demographics was administered through REDCap (Research Electronic Data Capture), a digital research data tool, during packed red blood cell transfusion visits. Parents of children receiving chronic transfusions, as well as adolescents and young adults receiving chronic transfusions, were enrolled between August 2018 and June 2019. Patients had to have a hematologic diagnosis requiring chronic transfusions, be receiving ICT, and be aged 12 years or older to complete the survey. Parents were required to have a child aged 24 months who met these criteria.

**Results:**

A total of 60 participants were included (median age 31.5, IQR 20-39 years; n=40, 67% female), with 29 (48%) being parents and 31 (52%) being patients. All parents and patients owned an electronic tablet, a smartphone, or both. The most endorsed mHealth app features among all participants included laboratory test monitoring (55/60, 92%), reminders to take iron chelation medication (50/60, 83%), and education about ICT (49/60, 82%). Parents’ most endorsed features included laboratory test monitoring (27/29, 93%) and education about ICT (25/29, 86%). Patients’ most endorsed features included laboratory test monitoring (28/31, 90%) and reminders to take iron chelation medication (28/31, 90%). There were no substantial differences between parents and patients in their preferences.

**Conclusions:**

Both parents and adolescents and young adults reported a strong interest in multiple mHealth app features. Participants provided valuable insights into optimal strategies and preferred app features for developing a multifunctional technology-based behavioral intervention to promote ICT adherence.

## Introduction

Iron overload is the major cause of morbidity and mortality in transfusion-dependent hematological disorders such as thalassemia [1]. One of the cornerstones of treatment for refractory anemia is packed red blood cell transfusions. Over time, transfusions can lead to excess iron accumulation in the heart, liver, and spleen, among other tissues, leading to a wide array of complications, including endocrinopathies, cardiomyopathies, and hepatic failure.

Iron chelation therapy (ICT) is essential for people with thalassemia and other hematological disorders requiring long-term red blood cell transfusion to minimize the risk of iron overload. Previous work has shown that adherence varies widely and is often suboptimal [2-5]. Suboptimal adherence to iron chelation medication is prevalent and has been associated with an increased risk of iron overload, resulting in increased morbidity, mortality, and health care use. Thus, interventions to improve ICT adherence are important to investigate and essential to improving morbidity and mortality.

Medication adherence is complex and multifaceted [6,7] Studies of adherence among patients taking iron chelation medication often report multiple factors contributing to low adherence, and it is difficult to compare and establish rates of adherence among patients taking iron chelation medication [2,3]. One potential avenue to address and improve adherence to medications is through the development of mobile health (mHealth) tools, that is, the use of mobile and wireless applications (eg, SMS text messaging, apps, wearable devices, remote sensing, and social media) in the delivery of health-related services [7,8]. The most recent (2021) Pew Research Center survey found that 97% of adult US residents owned a mobile phone, with 85% owning a smartphone [9]. Similarly, the vast majority of adolescents have access to digital devices, including smartphones (95%), and 97% of all adolescents report using the internet daily [10].

However, while the Pew Research Center surveys are designed to sample a population that reflects the demographics of the United States, they do not necessarily reflect the demographics of individuals with chronic medical conditions such as thalassemia and their families. There is a known “digital divide” in the United States, with disparities in access to technology correlated with a variety of factors, including household income, educational level, and geography [11]. It is important to ensure that pediatric patients, adolescents, and young adults receiving chronic transfusions, as well as parents, have access to technology when considering mHealth interventions.

Moreover, there is a variety of potential avenues and tools that can be integrated into mHealth interventions. Observational studies have shown that sharing health care experiences online can lead to decreased isolation, increased support, an exchange of coping strategies, and health care learning from shared experience [12]. There is also some evidence that both healthy adolescents and those with chronic health conditions who develop skills to monitor their symptoms and self-manage their health may experience improved outcomes in disease knowledge and adherence specifically through SMS text messaging and mobile phone apps [13-16]. A pilot study of a medication reminder app demonstrated the feasibility and potential usefulness of mHealth in adolescents and young adults receiving chronic transfusions [17]. In addition to access to mHealth and the efficacy of these features, it is important that mHealth interventions have high engagement. While adolescents in general are avid smartphone users, only 2% of adolescents report frequent use of an mHealth app [18]. Thus, it is crucial that mHealth interventions are designed with accessibility and engagement in mind.

Involving users in the early development process has been shown to promote engagement [19,20]. User-centered app design is a method of designing mobile apps that begins with a needs assessment followed by iterative cycles involving the intended end user. Apps involving end-user input throughout the development, testing, and dissemination process are more likely to be perceived by users as useful as well as easy to use [21].

Thus, this study was a needs assessment for an mHealth adherence app as the first step in a user-centered design process. First, we aimed to evaluate patients’ and parents’ access to technology-based (mHealth) interventions. Second, we aimed to assess interest in and preferences for an mHealth intervention to promote ICT adherence. We hypothesized that adolescents and young adults and parents of children receiving chronic transfusions would have high interest in an mHealth app and that adolescents and young adults and parents may have different preferences and priorities for mHealth. Ultimately, this study is the first step in developing an mHealth tool to promote medication adherence and optimize health outcomes among patients receiving chronic transfusions.

## Methods

### Recruitment

This was a cross-sectional, single-institution study. Participants completed a cross-sectional survey that was administered through REDCap (Research Electronic Data Capture; Vanderbilt University; a secure, web-based tool for research data capture used for collecting data and surveys) using tablet computers during packed red blood cell transfusion visits at a single institution. Eligibility criteria for patients included (1) having a hematologic diagnosis requiring chronic transfusions, (2) being on ICT, and (3) being aged 12 years or older to complete the survey. Parents were required to have a child aged 24 months or older who met these criteria. Potential study participants (parents of children receiving chronic transfusions and adolescents and young adults receiving chronic transfusions) were approached during transfusion appointments between August 2018 and June 2019. Data were collected on electronic tablets through REDCap supported by the Northwestern University Clinical and Translational Sciences Institute. This study aimed to accrue 50 to 100 patients based on feasibility and patient volume at our infusion center. By the end of the accrual period, a total of 60 participants were enrolled.

### Ethical Considerations

The institutional review board at the Ann and Robert H. Lurie Children’s Hospital of Chicago approved this study, and all procedures were conducted in accordance with the current version of the Declaration of Helsinki. Informed consent was obtained from all participants, and they were aware of their ability to opt out. Survey data were anonymous and did not contain identifying information. All participants were compensated with a US $25 gift card upon successful completion of the study survey.

### Study Measures

Our study instrument included 63 items assessing technology access, mHealth preferences, and demographics. These items were based on current literature investigating technology-based interventions and medication adherence among patients with chronic conditions in adult and pediatric populations based on previous studies conducted at our institution [6,16,22-24].

The technology access portion of the survey included 7 questions about access to electronic devices, as well as 8 questions about SMS text message and call limits and home or school internet signal strength. The mHealth portion of the survey included 8 yes-or-no questions, 1 rank-order question, and 6 multiple-choice questions that evaluated interest in general mHealth features and notification preferences, which have been previously reported in studies conducted at our institution [16,23].

### Statistical Analysis

Descriptive statistics for categorical data were reported in frequencies and percentages. Chi-square tests were run to determine the significant associations among variables and different subgroups. All tests were 2-sided, and a *P* value of <.05 was considered statistically significant. Statistical analysis was conducted using Microsoft Excel.

## Results

### Demographics and Technology Access

A total of 60 participants were included, with 29 (48%) being parents and 31 (52%) being patients (Table 1). The median age of the participants was 31.5 (IQR 20-39) years, and 67% (n=40) were female. The most common diagnoses included thalassemia (n=31, 52%) and sickle cell disease (n=21, 35%). Reported ICT medications varied, with 65% (n=39) taking deferasirox in oral formulation, 27% (n=16) taking deferasirox in tablet form, 8% (n=5) taking deferiprone, and 5% (n=3) taking deferoxamine. All participants’ characteristics are summarized in Table 1.

All parents and patients owned an electronic tablet, a smartphone, or both. Most parents and patients owned cell phones (28/29, 97% and 28/31, 90%, respectively), but tablet ownership was less pervasive (19/29, 66% and 10/31, 32%, respectively). Most parents and patients had unlimited plans for SMS text messaging (27/29, 93% and 25/31, 81%, respectively) and data (21/29, 72% and 21/31, 68%, respectively), as well as a fast home internet connection (24/29, 83% and 23/31, 74%, respectively).

**Table 1.** Participants’ characteristics and demographics as self-reported on a cross-sectional survey (N=60).

	Values
Age (y), median (IQR)	31.5 (20-39)
Age group, n (%)	
Parents	29 (48)
Patients	31 (52)
Adolescents (12-17 y)	10 (17)
Young adults (≥18 y)	21 (35)
Female sex, n (%)	40 (67)
Race or ethnicity, n (%)	
Asian	23 (38)
Black	16 (27)
Hispanic	4 (7)
White	17 (28)
Diagnosis, n (%)	
Thalassemia	31 (52)
Sickle cell disease	21 (35)
Other hematological disorders^a^	8 (13)
Iron chelation medication, n (%)	
Exjade (deferasirox)	16 (27)
Jadenu (deferasirox)	39 (65)
Ferriprox (deferiprone)	5 (8)
Desferal (deferoxamine)	3 (5)
Other medications, n (%)	
Hydroxyurea	1 (2)
Penicillin	13 (22)
Folic acid	3 (5)
Insulin	3 (5)
Multivitamins	4 (7)
Amiloride	3 (5)
Others^b^	16 (27)
Insurance, n (%)	
Private	34 (57)
Public or Medicaid	18 (30)
Unsure	3 (5)
None	5 (8)

a Other hematological disorders among participants included sideroblastic anemia (1/60, 2%), Diamond-Blackfan anemia (3/60, 5%), congenital dyserythropoietic anemia (2/60, 3%), Fanconi anemia (1/60, 2%), and pyruvate kinase deficiency (1/60, 2%).

b Other medications included ibuprofen (1/60, 2%), betamethasone ointment (1/60, 2%), risedronate (1/60, 2%), levothyroxine (1/60, 1.7%), hormonal replacement therapy (1/60, 2%), medroxyprogesterone (1/60, 2%), wellbutrin (1/60, 2%), venlafaxine (1/60, 2%), enalapril (1/60, 2%), hydrocortisone (1/60, 2%), hydroxychloroquine (1/60, 2%), oral contraceptive pills (1/60, 2%), aspirin (1/60, 2%), and unspecified medications (3/60, 5%).

### Interest in mHealth Features

All 8 proposed mHealth features were endorsed by over 50% (30/60) of the participants, including parents (15/29, 52%) and patients (16/31, 52%; Table 2). The median number of features endorsed by both parents and patients was 7 (IQR 6-8). The most endorsed mHealth app features among parents and patients included laboratory test monitoring (55/60, 92%), reminders to take iron chelation medication (50/60, 83%), and education about ICT (49/60, 82%). In particular, parents’ most endorsed features included laboratory test monitoring (27/29, 93%) and education about ICT (25/29, 86%). Patients’ most endorsed features included laboratory test monitoring (28/31, 90%) and reminders to take iron chelation medication (28/31, 90%). There were no significant differences between parents and patients in their preferences (Table 2).

**Table 2.** Frequency of participants’ reported interest in general mobile health features as assessed via a cross-sectional survey (N=60).

App feature	All participants, n (%)	Parents (n=29), n (%)	Patients (n=31), n (%)	*P* value
Medication reminders	50 (83)	22 (76)	28 (90)	.13
Medication log	45 (75)	23 (79)	22 (71)	.46
Positive feedback	47 (78)	20 (69)	27 (87)	.09
Adherence SMS text message prompt	48 (80)	21 (72)	27 (87)	.16
Social media	39 (65)	20 (69)	19 (61)	.53
Diagnosis education	45 (75)	23 (79)	22 (71)	.46
Medication education	49 (82)	25 (86)	24 (77)	.38
Laboratory test monitoring	55 (92)	27 (93)	28 (90)	.70

**Figure 1.**
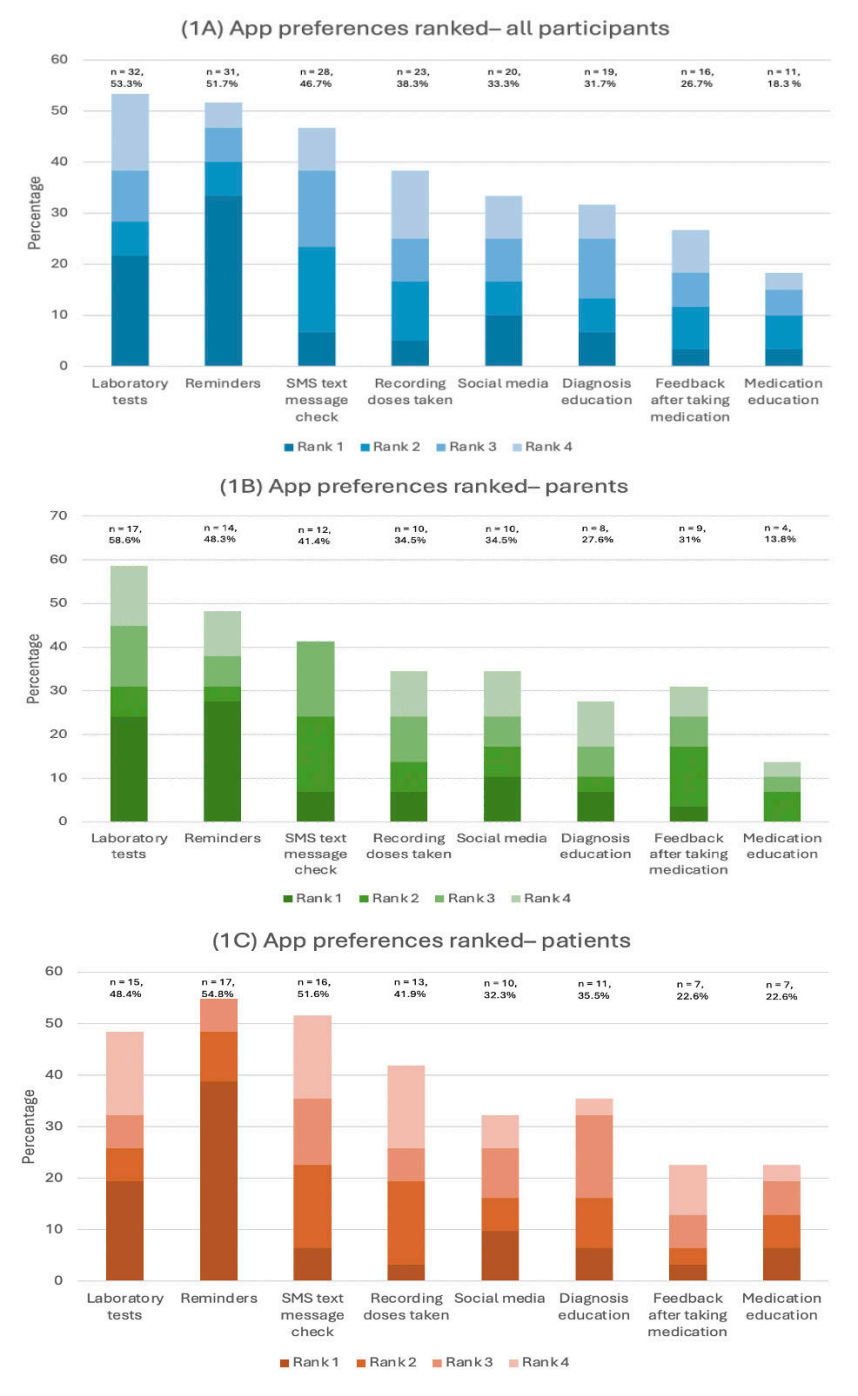
Cumulative ranked preferences for a thalassemia smartphone app among all participants (parents and adolescents and young adults) as ranked via a cross-sectional survey: (A) all participants, (B) parents, and (C) patients.

The cumulative ranking of the proposed smartphone app features among parents and adolescents and young adults is summarized in Figure 1. Medication reminders (8/29, 28%) were most frequently ranked as most important among parents, followed by the ability to review laboratory test results (7/29, 24%) and social media features (3/29, 10%). Patients also most prioritized medication reminders (12/31, 39%), followed by laboratory test results (6/31, 19%) and social media features (3/31, 10%).

## Discussion

### Principal Results

Our study aims were to evaluate patients’ and parents’ access to technology-based (mHealth) interventions and assess interest in and preferences for an mHealth intervention to promote ICT adherence. These findings highlight that parents and adolescents and young adults had high rates of technology access and reported strong interest in multiple mHealth app features, including features that specifically focused on medication adherence, education, and connection with the community.

Our participants’ high rate of technology access is comparable to that in reports of the general population and supports that mHealth interventions may be an accessible, useful tool to promote adherence [10,12]. Our study adds to the existing literature by demonstrating a strong interest in multiple mHealth app features by patients receiving chronic transfusions. Participants provided important insights into preferred app features for developing a technology-based behavioral intervention (mHealth app). These findings can help guide the development of an effective mHealth tool to promote ICT adherence for adolescents and young adults with thalassemia or other chronic transfusion–dependent conditions.

While there are some limited mHealth apps currently available, the strong interest from both patients and parents in mHealth features in our study suggests that there may be some features and tools that would be more beneficial and engaging to users than those that currently exist. This further highlights the importance of user-centered development in ensuring that these apps prioritize features that are of interest to the populations that are intended to use them. One international study in 2018 found that, although there are currently many mHealth apps available for use, they often do not match patients’ expectations due to lack of user-centered development [24]. Our study represents the starting point of user-centered design and assessment of interest in mHealth interventions and features among patients receiving ICT and their parents. User-centered design should continue throughout the phases of development, including creation, deployment, testing, implementation, and dissemination [21].

Of the app features highlighted in our study, there was particularly strong interest in laboratory test monitoring, medication reminders, and social media or virtual connections with other patients with transfusion-dependent illnesses. Of note, there was no statistically significant difference between the mHealth preferences of parents and patients surveyed, with a nonsignificant but slightly greater proportion of patients preferring positive feedback when taking medications compared to parents. Prior literature has often highlighted the ways in which barriers to adherence may vary from adolescence to adulthood and the ways in which adolescents may have unique preferences for mHealth interventions due to their relative use of technology compared to other age groups [25-27]. One possible explanation for the lack of discrepancy between the preferences of parents and patients is that most participants had interest in multiple mHealth features, with a median number of features endorsed of 7 out of 8. An mHealth tool with multiple features may be beneficial to both patients receiving ICT and parents.

This study builds on previous literature examining adherence interventions for patients receiving ICT [2-4,17]. In particular, a 2017 study highlighted a pilot intervention using a medication reminder app and demonstrated the feasibility and potential usefulness of mHealth in adolescents and young adults receiving chronic transfusions. Our study was focused more on the development stages of an mHealth intervention, particularly on obtaining user-centered feedback on which features might be preferred and promote adherence. Moreover, our study examined not only adolescents and young adults but also parents, who are also a potential user group for an mHealth intervention.

The results of this study are consistent with the literature on technology access and preferences among patients with other complex chronic conditions, including cystic fibrosis, diabetes, sickle cell disease, and acute lymphoblastic leukemia [16,23,28-30]. This further supports that an mHealth app with multiple features could be a promising tool to promote ICT adherence in patients receiving chronic transfusions.

### Strengths

Our study had several strengths. We were able to conduct a thorough evaluation of access to technology, assessing both access to multiple modes of technology and barriers to technology use, such as data plans and Wi-Fi access. Moreover, this study examined both patients’ and parents’ preferences for an ICT mHealth app, allowing us to effectively examine a group of all potential users of this mHealth intervention while also comparing preferences. Lastly, our patient population was diverse, both racially and ethnically and in terms of ICT medications used, as well as in insurance status.

### Limitations

There are several limitations worth noting in this study. First, this was a single-institution study with a relatively small sample size. While our survey items were not validated, they have been used in other published studies [16,22]. Additionally, as we adapted existing survey items, we did not provide an exhaustive list of potential mHealth interventions and app features. Finally, we did not survey our participants about prior experience with mHealth apps, which may have been helpful to note in participants’ perceptions of mHealth and preferences for mHealth features.

### Conclusions

In conclusion, parents and patients reported high accessibility to mobile technology. Overall, there was a high level of interest in mHealth interventions, as well as in features specifically intended to promote medication adherence. These findings support an interest in and need for the development of a user-centered mHealth intervention as a tool to promote medication adherence among patients with thalassemia and other conditions requiring chronic transfusions, as well as among their parents. Parents and patients reported similar preferences for mHealth features centered on medication reminders, education, and connection with the broader community of individuals requiring chronic transfusions and their families. In the future, continuing to center user experiences and feedback will be important in maximizing engagement with and utility of an app as an intervention to improve adherence and health care outcomes among patients receiving ICT.

Next steps may include developing and designing an app for an mHealth intervention integrating participant preferences. Throughout development and implementation, centering user experience will continue to be important. Future studies could then use participant feedback to assess the effectiveness of these tools in promoting medication adherence and optimizing health outcomes while also modifying the mHealth tools to be more effective among patients and parents of individuals receiving chronic transfusions.

## Abbreviations

ICT: iron chelation therapy
mHealth: mobile health
REDCap: Research Electronic Data Capture
